# Deformable microlaser force sensing

**DOI:** 10.1038/s41377-024-01471-9

**Published:** 2024-06-05

**Authors:** Eleni Dalaka, Joseph S. Hill, Jonathan H. H. Booth, Anna Popczyk, Stefan R. Pulver, Malte C. Gather, Marcel Schubert

**Affiliations:** 1 Centre of Biophotonics, SUPA, School of Physics and Astronomy, University of St Andrews, North Haugh, St Andrews, UK; 2 Humboldt Centre for Nano- and Biophotonics, Department of Chemistry, University of Cologne, Köln, Germany; 3 School of Psychology and Neuroscience, University of St Andrews, St Mary’s Quad, South Street, St Andrews, UK; 4 Present Address: Institute for Bioengineering of Catalonia, Barcelona, Spain

**Keywords:** Applied optics, Optical spectroscopy, Biophotonics

## Abstract

Mechanical forces are key regulators of cellular behavior and function, affecting many fundamental biological processes such as cell migration, embryogenesis, immunological responses, and pathological states. Specialized force sensors and imaging techniques have been developed to quantify these otherwise invisible forces in single cells and in vivo. However, current techniques rely heavily on high-resolution microscopy and do not allow interrogation of optically dense tissue, reducing their application to 2D cell cultures and highly transparent biological tissue. Here, we introduce *DEFORM*, *deformable microlaser force sensing*, a spectroscopic technique that detects sub-nanonewton forces with unprecedented spatio-temporal resolution. DEFORM is based on the spectral analysis of laser emission from dye-doped oil microdroplets and uses the force-induced lifting of laser mode degeneracy in these droplets to detect nanometer deformations. Following validation by atomic force microscopy and development of a model that links changes in laser spectrum to applied force, DEFORM is used to measure forces in 3D and at depths of hundreds of microns within tumor spheroids and late-stage *Drosophila* larva. We furthermore show continuous force sensing with single-cell spatial and millisecond temporal resolution, thus paving the way for non-invasive studies of biomechanical forces in advanced stages of embryogenesis, tissue remodeling, and tumor invasion.

## Abstract (web summary)

Characterising forces deep inside biological tissue is a challenging task. Here, we demonstrate that deformable biointegrated microlasers can sense nanoscopic forces with unprecedented spatio-temporal resolution in vitro and in vivo.

## Graphical abstract

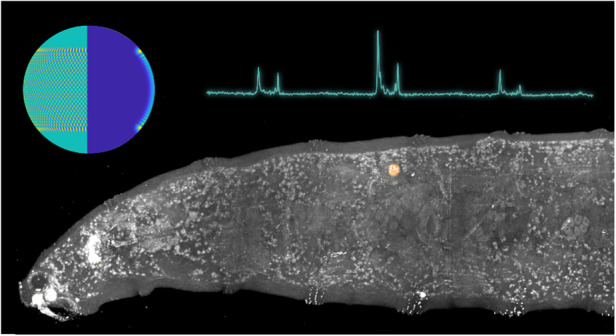

## Introduction

Advancements in optical and electron microscopy, as well as biochemistry, and genetics have revealed various factors that guide the development of large multicellular animals. Over the past two decades, mechanical forces have emerged as a key factor among these, and a number of sophisticated techniques to measure forces in 2D cell cultures and 3D tissue have been developed^1–3^. However, these methods rely almost exclusively on direct imaging of the deformation of either the biological sample itself or a support structure from which the mechanical force is subsequently calculated^4–8^. For example, injected ferrofluidic droplets^5,6^ or flexible polyacrylamide beads^7,8^ can measure mechanical stresses on the tissue scale in vivo but are ultimately limited by the ability to optically resolve the exact size of the droplets or 3D shape of the irregularly deformed beads, respectively. Alternatively, ultrasensitive force spectroscopy can be performed with optical or magnetic tweezers^9–11^. These techniques are able to resolve forces in the range of 0.1–100 pN, allowing to accurately measure forces of single motor proteins or the torsional stress stored in DNA strands as well as forces associated with cellular single receptor binding. Recently, in vivo trapping and manipulation of single cells has also been demonstrated^12,13^. However, dense tissue environments with high mechanical stresses or highly opaque samples remain challenging to investigate with optical or magnetic traps.

Consequently, the dependence on high resolution imaging or controlled optical trapping has so far limited the investigation of forces to samples with minimal absorption and scattering. Furthermore, these techniques often rely on high intensity laser scanning or trapping which can compromise the health of delicate biological tissues and has only moderate temporal resolution. Our current knowledge of biomechanical mechanisms is therefore deduced from investigations on a very limited number of animal models, namely the early stages of zebrafish and *Drosophila* embryos^4–7,14^, or other flat transparent structures like the *Drosophila* wing^15^.

Microscopic lasers have emerged as a powerful technique for single cell tracking^16–19^ and biomedical sensing^20^, including for the detection of proteins and disease markers^21–23^, the structural change of biomolecules^24^, and the sensing of contractions of heart cells^25^. Furthermore, in a proof-of-concept experiment, a droplet microlaser was injected into a single cell and the flattening stress the cell exerted onto it was estimated from analyzing the emission spectrum of the laser^17^. However, due to the lack of reliable calibration measurements and the limited range of biological samples that were studied so far, the potential of droplet microlasers for biomechanical measurements remains largely unexplored^26^.

To overcome the current limitations of conventional force measurements we have developed a microlaser-based method that can quantify the miniscule forces of single cells exerted deep within structurally intact, non-transparent tissue, while also covering a wide range of spatiotemporal scales. Our method is first validated by comparison to a gold standard technique and subsequently applied in two models widely used to study tumor growth and developmental biology. We believe that the development of new experimental designs for long-term integration of such microscopic force sensors will decipher the mechanical processes acting in the development of complex lifeforms and the progression and treatment of a broad range of diseases.

## Results

### Optical modeling and fabrication of droplet lasers

Micrometer-sized oil droplets can form high quality optical resonators, confining light by total internal reflection and thus supporting the formation of whispering gallery modes (WGM)^26,27^. By adding fluorescent dye to the oil, optical gain can be introduced which in turn allows WGM lasing under optical pumping with nanosecond laser pulses. The resulting droplet microlaser emission spectrum consists of intense and sharp lines with a regular pattern of transverse electric (TE) and transverse magnetic (TM) laser modes which represent the two orthogonal polarizations of the trapped light^28^ (Fig. 2b, g). The spectral position of these modes contains accurate information about the droplet, namely its diameter and bulk refractive index. In addition, the refractive index in the close proximity (~100 nm) of the droplet surface can be extracted^25^.

**Fig. 1:**
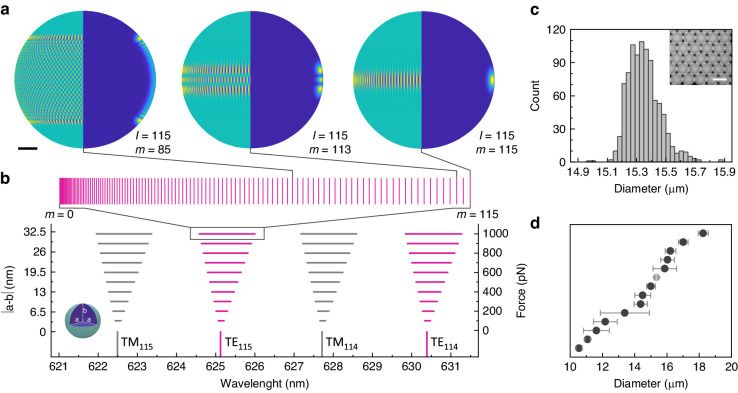
Principle of deformable microlaser force sensing. **a** Sketch of whispering gallery modes inside microscopic spherical droplets for three different azimuthal mode numbers *m* = 115, 113, and 85. Shown are the surface projections of the electromagnetic field (left half of the spheres) and the cross section of the modes inside the droplets (right half of the spheres). Scale bar, 2 µm. **b** Calculated mode splitting upon deformation of a 15 µm diameter spherical droplet into an ellipsoid with oblate geometry. Lifting of the mode degeneracy causes the single mode to split into broad bands. A zoomed-in view of the transverse electric mode TE_115_ shows that the approximately 1 nm wide band consists of 115 azimuthal modes. The inset shows an oblate ellipsoid with the equatorial semi-axis *a*, polar semi-axis *b*, and *b* < *a*. **c** Size distribution of droplets fabricated in a high throughput microfluidic chip. Inset: Fluorescence microscopy image of the fabricated droplets. Scale bar, 15 µm. **d** Droplet diameters and size distributions for batches of droplets that were fabricated under different flow conditions. Data are offset vertically for clarity. Error bars indicate standard error of the mean

The spherical geometry of a microscopic droplet supports a variety of WGM modes, characterized by a set of the three integer mode numbers: *l* ≥ 1 (angular momentum), *n* ≥ 1 (radial), and *m* ∈ [−*l*,*l*] (azimuthal)^28^. For *m* = *l*, the mode is located at the equator, while for 0 < *m* < *l* the modes expand increasingly towards the poles (Fig. 1a). In a perfectly spherical resonator, all 2*l*+1 azimuthal modes are degenerate, that is they have identical resonance energies and therefore cannot be distinguished in the spectrum. However, upon deformation of the sphere into an ellipsoid, this mode degeneracy is lifted, and each azimuthal mode then assumes a characteristic resonance energy that can be resolved as a splitting of the original peak in a high resolution emission spectrum of the droplet microlaser (Fig. 1b)^29–31^. Our DEFORM method correlates this force-induced laser mode splitting to the geometric shape of the ellipsoid. Combined with a careful characterization of the mechanical properties of the droplet, this allows the extraction of the force that causes its deformation. We show that deformations in the nanometer range can be resolved, enabling the measurement of sub-nanonewton forces with high accuracy. For example, for a droplet with a diameter of 15 µm and a surface tension of 5 mN/m, a 200 pN unidirectional force would cause a difference in the length of the ellipsoidal major axes of only 6.5 nm, which corresponds to an ellipticity *e* of ∼0.08%, yet causes a mode splitting of 285 pm, large enough to be readily resolved with a high-resolution spectrometer (Fig. 1b).

**Fig. 2:**
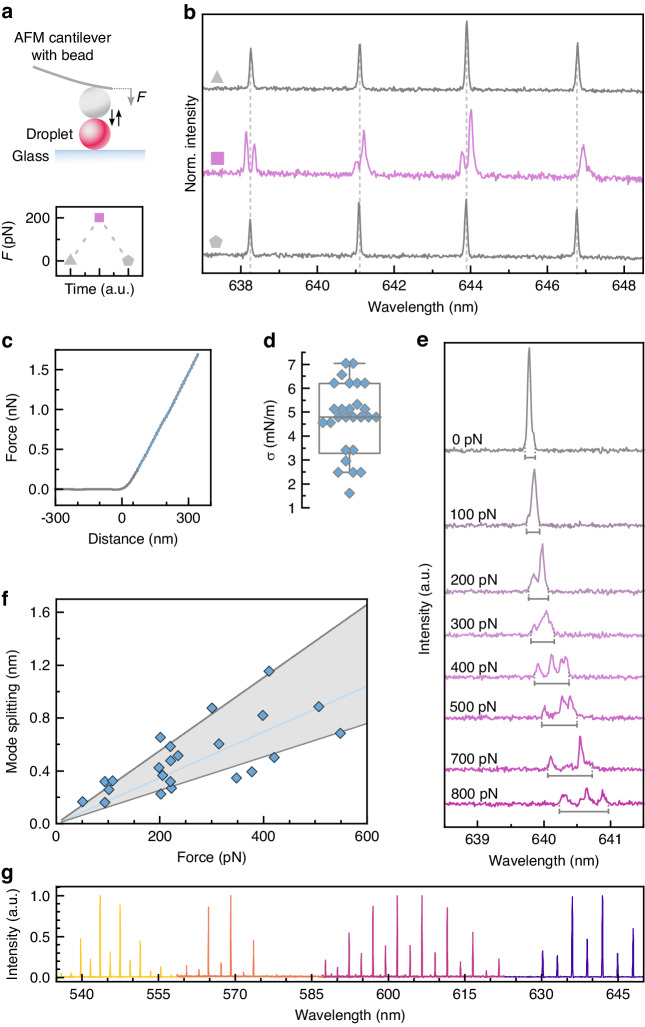
DEFORM reliably measures sub-nanonewton forces. **a** Schematic illustration of the deformation of single microlaser droplets by an atomic force microscope (top) and visualization of a typical push-and-release experiment (bottom). **b** Microlaser spectra detected before, during, and after the application of a 200 pN force with the AFM (symbols as in **a**). **c** Typical force-distance curve used to calculate the stiffness of the droplets. **d** Variation in stiffness for a batch of droplet microlasers (*N* = 27). Boxplot showing the median and standard deviation, while whiskers represent the 5^th^ and 95^th^ percentile. **e** Evolution of the microlaser spectrum under increasing applied force. The gray bars below each spectrum indicate the fitted mode splitting, i.e. separation in wavelength between the leading and trailing edge of the mode. All spectra are plotted on the same scale but vertically offset for clarity. **f** Mode splitting versus force applied by AFM. A linear regression analysis is used to obtain the correlation between laser mode splitting and external force (solid blue line). The gray area marks the error of the measurement used for further calculations of the force. **g** Lasing spectra of undeformed microlasers doped with 4 different fluorescent dyes, C545T, BODIPY, Nile Red, Rhodamine B (left to right)

To implement DEFORM, we fabricated microdroplets with controlled size and surface properties using a high throughput microfluidic system (Fig. 1c, d). By working with high refractive index oils, we ensure that droplets with diameters down to ∼10 µm readily generate laser emission. Larger resonators can also be used, but the decreasing free spectral range limits the range over which splitting of individual modes can be resolved. In this study, we therefore opted for diameters between 10 and 20 µm to balance resonator size and sensitivity. In addition to the size, the surface tension of the droplets can be precisely adjusted by coating the droplets with different surfactant molecules which also stabilizes the droplets inside biological tissue^31^. Importantly, the biocompatibility of the high refractive index oils and surfactant molecules were assessed by cell proliferation assays which showed no negative effect (Supplementary Fig. S1). Additional functionalization is possible by coating the droplet surface with molecules that either facilitate or hinder cell adhesion^32^.

### Droplet calibration and force calculation

We first describe validation and calibration measurements that demonstrate the ability of DEFORM to extract absolute mechanical forces. To characterize the mechanical properties of the microlaser droplets, individual droplets were deformed under a well-defined force applied by an atomic force microscope (AFM, Fig. 2a) while simultaneously detecting the microlaser emission. Starting with the resolution-limited spectrum of the undeformed droplet, application of an external force causes a distinct mode splitting ${\sigma }_{\text{split}}=|{\lambda }_{l,m=0}-{\lambda }_{l,m=l}|-{\delta }_{0}$ of the previously degenerate azimuthal modes into several sub-modes, where *δ*_0_ is the peak width of the undeformed droplet (i.e., the resolution of the spectrometer used), and $|{\lambda }_{l,m=0}-{\lambda }_{l,m=l}|$ is the fitted distance between the leading and trailing edge of each split mode, which we assume corresponds to the lowest and highest order azimuthal modes^33^. Removing the external force yields the original spectrum, showing that the droplet relaxes back into the spherical geometry (Fig. 2b). From the AFM force-distance curves we extracted the surface tension of individual droplets, finding a mean of 4.5 mN/m for droplets produced with 0.6 mM of Tween 20 surfactant (Fig. 2c, d), in good agreement with pendant droplet tensiometer measurements (Supplementary Fig. S2). We then systematically increased the force applied by the AFM (Fig. 2e) and observed a more pronounced mode splitting that scaled linearly with the applied force (Fig. 2f), consistent with the analytical calculations (Fig. 1b). The smallest detectable peak splitting occurs at an applied force of 50 pN, mainly limited by the optical resolution of the spectrometer. Here, we note that the split spectra typically contain a very complex structure of sub-modes which we attribute primarily to the different scattering characteristics of the modes with varying azimuthal mode number.

Next, we describe our model to extract absolute forces from the recorded droplet spectra. Starting with estimates of the droplet size based on the fitted free spectral range^28^, the geometrical parameters of the droplet ellipsoid are extracted by analyzing the mode splitting separately for two subsequent angular mode numbers for both, TE and TM, polarizations (see Methods). The resulting geometry, expressed by the eccentricity *e* of the droplet, can then be averaged to reduce the effect of small variations in the width of the split modes, although we generally observe that the splitting is very consistent between modes with different angular momentum mode numbers. The eccentricity of the droplet is connected to the applied force via^34^

1 $$F=3\pi \gamma a\left[{\left(1-{e}^{2}\right)}^{x}-{\left(1-{e}^{2}\right)}^{y}\right]$$

Where, *a* is the equatorial semi-axis, *b* is the polar semi-axis, *γ* is the surface tension, and *x* = 1/6, *y* = 1/2, ${e}^{2}=1-\frac{{b}^{2}}{{a}^{2}}$ for an oblate, or *x* = −1/2, *y* = −1/6, ${e}^{2}=1-\frac{{a}^{2}}{{b}^{2}}$ for a prolate geometry. An error analysis that includes droplet size, external force, and emission wavelength suggests that the total error of the extracted force is below 3% for a wide range of parameters (Supplementary Fig. S3).

Finally, DEFORM can be adapted to operate across the entire visible and NIR spectrum, simply by changing the fluorescent dye that provides optical gain to the droplet lasers (Fig. 2g).

### Depth-resolved spectroscopic force sensing in large tumor spheroids

The microlaser droplets presented here are designed to resolve miniscule forces exerted by intracellular or intercellular interactions or forces in 3D cellular environments and small animals. As an example, we observe the variation of intracellular forces between migrating and resting cells which shows that the method is sensitive enough to perform force measurements with single cell resolution (Supplementary Fig. S4).

**Fig. 3:**
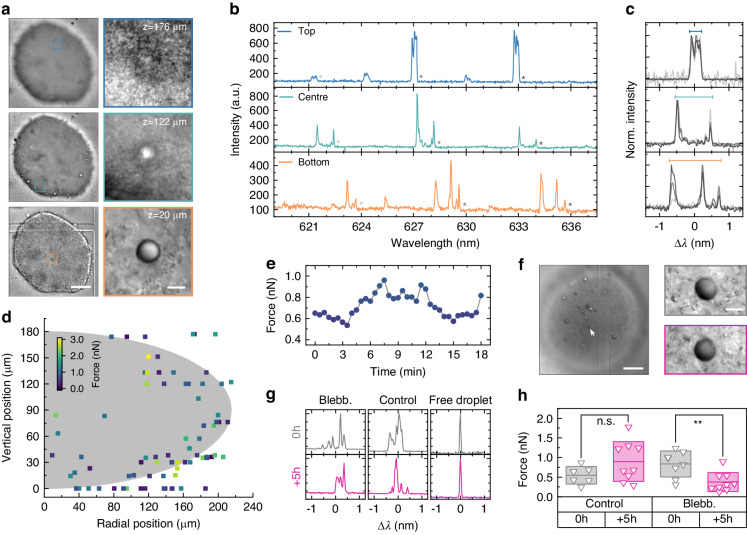
3D force sensing in tumor spheroids. **a** Left: Differential interference contrast (DIC) microscopy of a 3D tumor spheroid, imaged at 3 different focus planes, at bottom (orange), middle (mint), and top (blue). Scale bar, 100 µm. Right: Contrast enhanced magnifications of regions with a microlaser droplet for each plane. Scale bar: 10 µm. **b** Lasing spectrum of the droplets in the magnified regions in **a**). **c** Overlays of 3 split modes marked with asterisks in each panel in **b**. The colored bars indicate the fitted peak splitting. Modes in each panel have the same polarization. **d** Overview of measured forces for *N* = 5 spheroids. The gray area indicates the average size of the spheroids (with semi-axes *a* = 90 µm, *b* = 210 µm), showing the approximate position of the droplets inside the spheroids. **e** Time-lapse force measurement inside a tumor spheroid. **f** (left) DIC microscopy image of a multicellular spheroid treated with blebbistatin. Scale bar, 100 µm. (right) Magnified images of the droplet microlaser marked by an arrow in the main panel before (gray) and after (violet) blebbistatin treatment. Scale bar, 10 µm. **g** Representative emission spectra of droplet microlasers inside spheroids treated with blebbistatin (left) and an untreated control (center) at the start and after 5 h. Also shown are spectra of free droplets outside the spheroid. **h** Statistical analyses of the effect of blebbistatin. Boxplot showing the mean and standard deviation while a two-sample t-test was used for statistical analysis

A key advantage of DEFORM is its ability to quantify forces without the need to image the microlasers. This enables measurements deep inside large cellular structures and within living tissue which are generally not accessible by imaging due to strong scattering and absorption of light. We demonstrate this by characterizing the distribution of forces inside multicellular spheroids with ∼400 µm diameter that were formed by a cell line model for head and neck squamous cell carcinoma (UM-SCC-1, Fig. 3a). Lasing spectra with high signal-to-noise ratio are detected throughout the entire spheroid (Fig. 3b). Despite the complex shape of the spectra, overlaying three subsequent angular momentum mode numbers of the same polarization clearly shows that the mode splitting can be extracted reliably and independent of the depth or deformation of the droplets (Fig. 3c). Furthermore, our analysis routine can also extract the droplet deformation if one of the two polarizations is completely suppressed (typically this occurs for the TM modes which have lower Q factors) because the effect of mode splitting is so dominant that it is not necessary to know the exact angular momentum mode numbers. Scanning the entire volume of large tumor spheroids reveals a complex 3D ‘force-scape’ with forces ranging from several hundred pN to a few nN depending on the location of the droplet. For the UM-SCC-1 spheroids investigated here, we do not observe a systematic variation in force in either radial or vertical direction across the spheroid (Fig. 3d). This is consistent with the absence of a necrotic core or other structural features. Interestingly, tracking of individual droplets reveals dynamics within the spheroids that show force variations of up to 50% on the time scale of only a few minutes (Fig. 3e), demonstrating that the highly dynamic cellular environment of multicellular spheroids induces significant fluctuations in their biomechanical properties (Supplementary Video 1).

To determine whether our method can detect physiologically relevant changes in spheroid biomechanical properties, we next investigate the response of the droplet microlasers to inhibition of cellular contractility. Imaging of a droplet microlaser at the center of a multicellular spheroid shows no visual change of the droplet before and after the treatment with the myosin-II inhibitor blebbistatin (Fig. 3f). However, the emission spectrum reveals a statistically relevant decrease in mode splitting (Fig. 3g) and the corresponding average force at 5 h after addition of blebbistatin (Fig. 3h). By contrast, the control sample shows a small increase in force over the same time. Droplets located outside the spheroids show a resolution limited spectrum (Fig. 3g), demonstrating that droplets in a load-free environment assume a perfectly spherical shape.

### In vivo force sensing in *Drosophila* larva

Our work in tumor spheroids shows that DEFORM can measure forces in 3D structures. Next, we set out to determine whether it can be used in intact living animals. The forces that act on the internal tissue and organs of animals are difficult to detect due to their fast dynamics and the challenging heterogenous optical characteristics of live animals. Up to now, measurements of internal force-scapes inside intact animals are therefore extremely limited and where available, are often based on methods with very coarse spatial and temporal resolution (e.g. aspiration or indentation-based methods)^35^.

*Drosophila* larvae are soft-bodied, semi-transparent organisms with a highly complex internal structure where internal organs and muscles with varying degrees of opacity are constantly moving. Furthermore, networks of air-filled trachea pervade all tissues down to the level of single cells, causing strong light scattering inside the animal. Because of these challenging optical properties, internal measurements of forces in this popular model organism have not been possible so far, despite rapid progress in other animals or in *Drosophila* embryos^4,6,7^. Late-stage *Drosophila* larvae therefore represent an ideal testbed for assessing the performance of DEFORM in live animals.

**Fig. 4:**
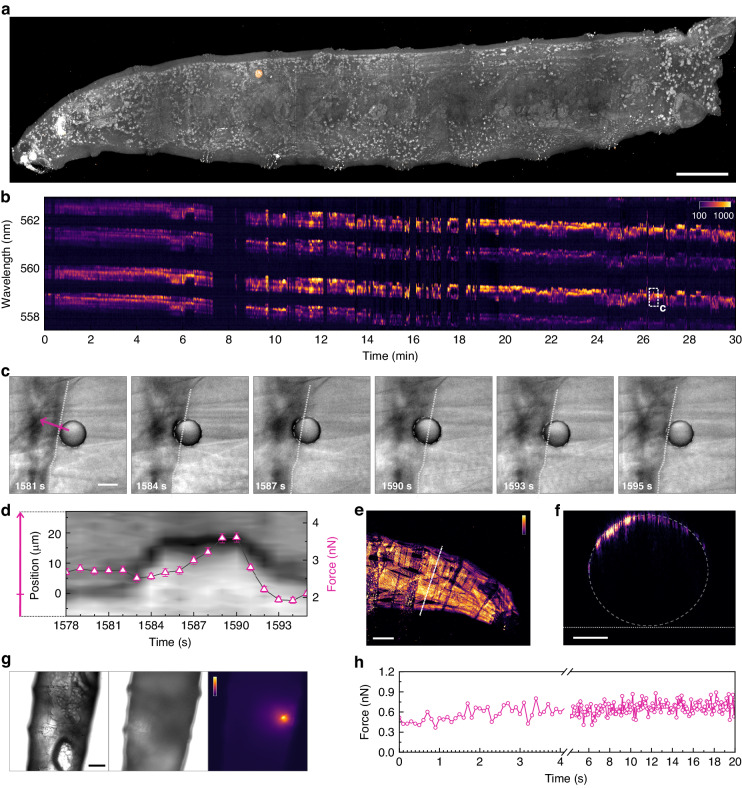
In vivo force sensing in optically complex *Drosophila* melanogaster larvae. **a** Fluorescence lifetime microscopy image of a 3rd instar *Drosophila* larva and an injected droplet microlaser. The tissue autofluorescence (gray) and the droplet microlaser (orange) are shown. Scale bar: 200 µm. **b** 30 min time lapse spectroscopy with 1s temporal resolution of a microlaser inside a *Drosophila* larva. The contour map shows the spectral intensity plotted on a logarithmic scale. Gaps in the measurement are due to manual readjustments of the microscope stage to keep the droplet microlaser inside the field of view (Supplementary Fig. S7). **c** DIC microscopy images from the time window highlighted in **b** (see also Supplementary Video 2). Scale bar: 10 µm. **d** Kymograph along the pink arrow drawn in **c** overlaid with the extracted force (pink, right axis). The position of the interface between microlaser and cuticle in the first frame is indicated by a tick mark on the pink arrow. Error bars represent the cumulated maximum uncertainties in the force value. **e** Maximum intensity projection of top-view multiphoton fluorescence microscopy of a *Drosophila* larva with GFP labeled musculature. Scale bar: 100 µm. **f** Cross-sectional view along the dashed white line shown in **e**. The white dashed line shows the approximate outline of the larva while the white dotted line indicates the position of the substrate. Scale bar: 100 µm. **g** Bottom-view transmission microscopy images focussed on the bottom (left) and top (middle) of a *Drosophila* larva. Fluorescence microscopy for the same top plane, revealing a highly blurred image of the injected microlaser droplet (right). Scale bar: 100 µm. **h** in vivo high-speed force transient with sub-nanonewton resolution measured at 10 Hz inside the larva shown in **g**

Droplet microlasers were injected into anesthetized 3rd instar larvae with thin microneedles attached to a custom designed pressure injection system. This minimally invasive technique allows targeting of specific parts of the animal (Fig. 4a). Injected droplets were monitored for several days to assess stability and location. We find that microlasers maintained stable size and optical properties, can traverse multiple internal segments, and have no negative effect on the development of animals. Single droplets could be imaged repeatedly over days and different developmental stages (Supplementary Fig. S5), and continuous measurements were acquired for ∼30 min (Fig. 4b). As observed before, all laser modes show a synchronized and well-defined broadening despite variations in the intensity distribution of the split modes. Forces varied dynamically as droplets moved from one region to the next showing variations from 3.2 nN to 7.5 nN (10–90 percentile) with a mean value of 5.5 nN (Supplementary Fig. S6). In some cases, microlasers became embedded in the larval muscles arrayed on the inside of the cuticle, causing acute deformations and resulting fluctuations in force. From the increase in bright field contrast, we identified an event where the droplet is actively pushed into a cuticle band (Fig. 4c, Supplementary Video 2). The microlaser response reveals that the required force increases steadily (Fig. 4d), possibly due to increasing resistance of the tissue with increasing indentation, demonstrating the high resolution with which such events can be characterized.

We also explored the maximum depth at which DEFORM measurements can be performed and compared this to state-of-the-art two-photon (2P) microscopy of the fluorescently labeled musculature (Fig. 4e). As visible in the cross-sectional view (Fig. 4f), only the outermost band-like muscle structures can be imaged although larvae have muscles throughout their entire body. In contrast and despite the presence of opaque cuticle, muscles, and other tissues, emission from the injected droplet laser was reliably detected and analyzed for depths of up to 300 µm and even when the pump beam passed through the entire animal (Fig. 4g). In this configuration, we further decreased the integration time to improve the temporal resolution. A fast time lapse measurement taken at 10 Hz displays force fluctuations on a sub-second time scale as well as a steady increase in force from about 0.4–0.8 nN over the course of 20 s (Fig. 4h).

## Discussion

The dynamic interplay of mechanical forces with cellular and genetic processes shapes how organisms grow and function. Forces at cellular levels have been widely studied in multicellular 3D structures like organoids and early-stage embryos. However, when 3D cell aggregates reach a size of roughly 100–200 µm, they typically become non-transparent, rendering the available techniques to analyze and manipulate their biomechanical properties ineffective. Recent benchmark studies have performed in vivo imaging studies on the 10-somite stage zebrafish embryo^36^, the first few hours of the *Drosophila* embryo development^37,38^, and sections of mouse embryos up to somite stage E8.5 d.p.c.^25,39^, even though in the latter case only selected, well accessible organs were investigated. Similarly, in vivo optical trapping experiments are so far limited to fully transparent samples and have been mostly performed in early-stage zebrafish enbryos^10,12,13^. In addition to their fundamental role in embryogenesis, biomechanical parameters like tissue stiffness, pressure, and forces are also severely altered in disease or aging^40^, adding further urgency to the need to develop techniques that can non-invasively monitor physical and mechanical properties of tissues in vivo.

DEFORM offers a new approach to quantify cellular and organ-scale forces across several orders of magnitude with exceptional temporal resolution. To validate the absolute scale of the extracted forces, we have used gold-standard AFM measurements to evaluate the optical response of individual droplets and have developed a simple optical model that allows to extract the eccentricity of the flexible microlaser droplets. Based on the known deformation of the microlasers, the applied force can then be directly calculated. Importantly, to extract the absolute force, DEFORM does not rely on any microscopy data; instead, it is entirely based on spectroscopic information. This has the advantage that microlasers can operate deep inside scattering tissue, beyond the depth of conventional fluorescence or multiphoton microscopes, which has allowed us to determine the scale and the dynamics of forces inside two widely used biological model systems.

Applying DEFORM to analyze tumor spheroids, we found that we can characterize the force-scape throughout the entire 3D multicellular spheroid. Considering that the microlasers are comparable in size to cancer cells, the measured forces are representative of effective cell-to-cell forces. Interestingly, the values measured by DEFORM are smaller than values extracted from techniques that analyze deformations of the extracellular matrix, for both, single cell and collective spheroid forces^41^, although we note that a different cell type was used in our study. This indicates that forces within spheroids can be considerably different compared to those exerted to the extracellular matrix around the spheroids.

In *Drosophila* experiments, we were able to observe the rapid movement of segmental muscles around the denticle bands that deformed the lasers while being pushed into an internal membrane. The peak force also coincides with the buckling of adjacent trachea, showing that the laser is actively pushed into the membrane by a muscle contraction. From multiphoton fluorescence microscopy, we estimate that the imaging depth at this larval stage is significantly below 100 µm. Consequently, only the upper half of the outermost muscles can be imaged in these animals, providing severe limitations for any force measurement technique that is based on imaging. Fast Fourier transfer (FFT) of force traces and analysis of spectrograms revealed no obvious rhythmic activity at any given frequency. However, we expect that with more targeted injections into regions of interest, and by implementing spectroscopic acquisition rates of up to 100 kHz^42^, DEFORM will help to shed light on highly dynamic processes, including myocardial organogenesis^43^, tissue contraction mechanics^44^, and ultrafast contraction events^45^.

Compared to existing force sensing technologies, DEFORM operates in a regime that makes it ideal to further investigate tissue scale mechanical stresses and interactions. The demonstrated sensitivity of about 50 pN is small enough to resolve single cell mechanical forces while at the same time the tunable interfacial tension of the droplet allows to monitor deformations inside dense microenvironments and living animals. Combining DEFORM with e.g. optical traps might also be used to guide droplet microlasers to specific parts inside the animals or to actively deform the droplets to measure external restoring forces^31,46^. However, the unique advantage of DEFORM over existing force sensing techniques is the direct spectral readout of mechanical forces independent of any additional structural or imaging information, allowing to perform experiments under conditions where it is otherwise currently not possible to obtain any precise force reading. Therefore, the potential applications to investigate mechanical processes in vivo include a wide range of animals and plants, most importantly those that are non-transparent^47^.

To conclude, we developed a novel opto-mechanical tool that enables in situ, label-free, and non-contact tracking of mechanical force at cellular and tissue-scale levels with microscopic spatial and real-time temporal resolution. Beyond this, our novel platform will be broadly applicable as it provides unique cell-microenvironment metrics to relate mechanical interaction to cell behavior.

## Materials and methods

### Droplet fabrication

Droplet microlasers were produced from a high refractive index (*n* = 1.67) polyphenyl-based oil (Santolight SL5267, Santolubes Inc.) that was doped with a red fluorescent dye (Nile red, 5 mM). Alternatively, for experiments with *Drosophila* melanogaster, a polyphenyl-based oil (Santovac 5, Santolubes Inc.) with a slightly lower refractive index (*n* = 1.63) was used. Santolight SL5267 and Santovac 5 are non-toxic and non-hazardous substances according to the material safety data sheets. The Santovac 5 droplet microlasers were doped with 5 mM of the Coumarin dye 10-(benzo[d]thiazol-2-yl)-1,1,7,7-tetramethyl-2,3,6,7-tetrahydro-1H-pyrano[2,3-f]pyrido[3,2,1-ij]quinolin-11(5H)-one (C545T, Luminescence Technology). For both oils, the dye concentration was optimized to yield the lowest lasing thresholds. We further observed that C545T has significantly improved long-term stability compared to Nile Red in biological environments and we therefore recommend C545T for experiments that span several days.

The droplet microlasers were fabricated in two different ways depending on the biological system. For single cells and multicellular spheroids, droplets were fabricated using a microfluidics setup to produce large numbers of highly uniform droplets. Aqueous Tween 20 at a concentration of 0.6 mM was used as a surfactant to stabilize and store the droplets. The surfactant also reduced the surface tension of the microlasers. A focussed flow geometry chip was used (00935, Micronit) which comprises a 10 μm wide nozzle. The continuous phase during production of the droplet was an aqueous Tween 20 solution. The disperse phase consisted of the oil/dye mixture. The flow in each channel was controlled using a custom-built pressure system that operated between 0 and 2000 mbar. Droplets were produced by keeping both channel pressures equal between 800 and 1200 mbar allowing to tune the droplet size between roughly 10–20 μm.

For experiments in *Drosophila* larva, droplets were fabricated by directly injecting the oil/dye mixture into the animal using a custom microinjection system. Injections were controlled by a custom microcontroller that produced pressure pulses of nitrogen at 6 bar for 200 ms through a freshly pulled (PC-10, Narishige) and bevelled (BV-10-D, Sutter) glass needle. The diameter at the base of the bevelled needle was about 3 μm, producing droplets between 15 and 30 µm. Bevelling was found to improve injection precision and minimizes injury and stress to the larva. During injections, the needle was controlled using a 3-axis mechanical micromanipulator (MMJL, WPI) and guided by the image of a stereo zoom microscope (SMZ18, Nikon).

### AFM measurements

Controlled deformations of single droplet microlasers were performed using an atomic force microscope (FlexAFM, Nanosurf) which was installed on the inverted microscope that was used for the lasing experiments, allowing simultaneous optical and mechanical characterization of the droplets. For indentation, a 17 µm glass sphere was glued to the tip of a soft cantilever with nominal stiffness of *k* = 0.01 N/m (qp-SCONT-20, Nanosensors). Cantilevers were calibrated prior the bead attachment using the thermal method. To stabilize the droplet microlasers on the glass substrate hydrogen bonds between the hydroxyl groups of Tween-20 on the droplet surface and the glass substrate were induced by lowering the pH of the PBS solution from 7 to 6^48^.

In a typical experiment, the cantilever was approaching the droplet with a speed of 1 µm/s until a set force value was reached. This set value was then kept constant for 10 s during which 20 lasing spectra were acquired which allows to extract averaged lasing characteristics under the applied force. Lasing spectra before and after droplet compression were also recorded. To test the appearance of viscoelastic effects on the droplet lasers, we performed experiments with mechanical creep (200 pN applied load for either 10 s or 5 min) and different approach velocities (1, 10 and 100 µm/s). No significant viscoelastic effects were observed in these experiments.

Measurements of the surface tension of microlasers were performed with a maximum force of 1 nN. The acquired force-distance curves were corrected for any linear shifts, and the contact point was set as the origin of the x-axis (distance). The data is then linearly fitted to extract the surface tension which is equal to the slope of the curve.

### Cell culturing

Cells of the head and neck squamous cell carcinoma (HNSCC) cell line UM-SCC-1 were provided by T. Carey from the University of Michigan. The cells were cultured in Dulbecco’s modified Eagle’s, phenol red-free medium (DMEM; 31053-028, Gibco), supplemented with 10% fetal bovine serum (FB-1001, Biosera), 1% minimum essential medium, nonessential amino acids (11140-035, Gibco), 1% GlutaMAX-I and 1% penicillin-streptomycin (15140122, Gibco).

To form 3D tumor spheroids, the hanging drop technique was applied. Briefly, methylcellulose (4000 cP, m-0512, Sigma Aldrich) was mixed with a suspension of UM-SCC-1 cells at a 1:4 (methylcellulose:cell medium) ratio and drops of 15 µl were formed on the inner side of the lid of a petri dish. The lid was flipped, and the hanging drops were incubated for 16–19 h at 37 °C and 5% CO2. The drops were then collected and transferred into a vial with cold media (approximately 200 µl). After the spheroids sedimented, the supernatant medium was exchanged with a mixture of 6 mg/ml basement membrane (3433-005-02, Cultrex) and 1.4 mg/ml collagen-I (354249, Corning HC). To incorporate droplet lasers into the tumor spheroids, 30–50 µl highly concentrated droplet solution were collected in an Eppendorf vial. After the droplets sedimented, the supernatant medium was removed, and the droplet microlasers were added to the cell solution. This mixture was then used with the hanging drop method as described above which results in a random distribution of droplets inside the spheroids. Adding extracellular matrix proteins (e.g. basement membrane matrix) to the cell/droplet mixture can increase the number of droplets that are incorporated in the spheroids.

To investigate if the measured microlaser peak splitting is a result of actomyosin contractility inside the cancer spheroids, cell contractility was inhibited by adding blebbistatin. The droplet position together with one laser spectrum was acquired at least 1 h before treatment with 50 µM blebbistatin. To avoid the formation of blebbistatin crystals, the blebbistatin solution was warmed to 37 °C before adding it to the sample. A sample with 50 µM DMSO (Sigma Aldrich) served as the control. Both control and blebbistatin samples were then incubated for 30 min, after which lasing experiments started. Laser spectra were measured for the same droplets tested before blebbistatin or DMSO addition and tracked over a duration of 5 h, acquiring one spectrum per microlaser every hour. Furthermore, to test whether blebbistatin treatment causes any effect on the lasing performance of the droplet lasers, free droplet microlasers were measured under both conditions.

Cell proliferation assays were performed for four different cell types, including primary rat aortic smooth muscle cells, 3T3-NIH cells, primary small intestine mouse endothelial cells, and primary human umbilical endothelial cells. Overall, 9 different conditions were tested, including two oils, four surfactant concentrations, and one control sample that contained no microlasers (Supplementary Fig. S1). At the start of the experiment, 1000 cells of each cell type were seeded separately into the wells of a 12 well plate culture dish. To each of these wells, 1000 droplets were added (except for the control sample that did not contain any droplet microlasers), and ten differential interference contrast microscopy images were taken from each well at 24 h intervals. The number of cells at each field of view was measured and the doubling time extracted from the gradient of the logarithm of the number of cells per field of view plotted over time. No statistically significant difference between the control sample and any of the droplet-containing samples was found, demonstrated by p-values larger than 0.05 (Kruskal-Wallis test).

### Detailed injection routine for *Drosophila* larva

Droplet microlasers were injected into the inter-visceral space of 3rd instar *Drosophila* melanogaster larvae. The injection was made in the middle of each animal, approximately at segments A2-A5. Bevelling of the microneedles greatly improves viability of the larvae due to a very clean and small injection site although no systematic study of the effect of droplet injection on the viability of the larvae has been performed. Larvae were anesthetised using non-direct exposure to diethylether for 2 min. Anesthetisation ensured that the animals were not motile during the droplet injection and subsequent measurements. During the 30 min measurement, movement of the internal organs of the animals were observed and the droplet moved freely within the inter-visceral space while the animal was still under the influence of the anesthetic and not fully motile. The movement of the microlaser through the animal was tracked manually by repositioning the animal in the fixed position of the pump laser.

To identify the depth limit of the microlaser experiments, droplet microlasers were injected into the dorsal side of 3rd instar wild type *Drosophila* larvae as close to the skin as possible. Subsequent excitation and read out were then performed from the ventral side. The distance in focal planes (measured with the internal sensor of the z-axis motor drive of the Nikon TE2000 microscope) between the denticles on the ventral side of the animals and the focal plane of the droplet microlaser (judged by the maximum signal intensity) is used to quantify the depth of the microlaser. Lasing spectra were taken at a maximum depth of 301 µm in a single-pulse detection scheme were the pump laser repetition rate and spectrometer frame rate were both set to 10 Hz. Here, the detection rate is only limited by the repetition rate of the pump laser which could be further increased.

### Multiphoton microscopy

Two photon microscopy images were taken of a 3rd instar larva that expressed green fluorescent protein in its muscles (ZCL::eGFP) to compare the imaging depth achievable by a state-of-the-art deep tissue imaging method compared to the droplet microlaser (Fig. 4e, f). Measurements were performed on an upright multiphoton microscope (Leica TCS SP8 MP-OPO, Leica Microsystems), equipped with a tunable ultrafast laser (Chameleon Vision II, Coherent), and an air objective (HC PL FLUOTAR 10×/0.3 DRY). The excitation laser was tuned to 940 nm and the emission was filtered with a bandpass filter (center 525 nm, width 50 nm) and detected with a non-descanned HyD detector.

Fluorescence lifetime imaging under multiphoton excitation was performed to image the injected droplet microlaser (Fig. 4a). Measurements were performed on an inverted multiphoton microscope (Leica Stellaris 8, Leica Microsystems), equipped with DIVE and FALCON units as well as a water immersion objective (HCX IRAPO L 25x/0.95 WATER). The tunable ultrafast excitation laser was set to 1045 nm, and the variable emission filter was set to 584–667 nm. Fluorescence lifetime phasor separation was used to split the channels, showing two distinct lifetime populations that belong to the autofluorescence (fast component) and the fluorescent dye inside the droplet microlaser (slow component).

### Microlaser experiments

Microlaser experiments were performed on a modified inverted research microscope (TE2000, Nikon) which has been described in detail previously^25^. Briefly, a tunable nanosecond pulsed pump laser is coupled into the microscope by a removable dichroic mirror that is located between the fluorescence filter cube turret and the microscope objective. An iris is placed in the optical path before the objective in an image plane that is conjugated to the focal plane of the sample to allow variation of the excitation area. This configuration creates a collimated rather than a focussed beam, allowing to excite the entire droplet microlaser. The light from the microlaser is then collected by the same objective and directed to the side port of the microscope. From here, a pair of achromatic relay lenses transfers the image to either a fast sCMOS camera (OrcaFlash 2, Hamamatsu) or a spectrometer consisting of a high-resolution spectrograph (Shamrock 500i, Andor) and an emCCD camera (Newton BV970, Andor). The optical resolution of the 1200 lines/mm grating was about 50 pm. Imaging and spectroscopy can be performed simultaneously by inserting a dichroic mirror in the detection path, and by using separate spectral bands for differential interference contrast imaging and microlaser spectroscopy, respectively.

For the time-tracked lasing experiments in *Drosophila* larvae, spectra were acquired every second over a period of 30 min. For each spectrum, integration over 4 pump pulses was performed and the pump source was an optical parametric oscillator (Opotek) tuned to 480 nm, with a repetition rate of 5 Hz, and a pulse width of about 7 ns.

### Spectral analysis

The droplet microlasers are modeled as having a non-spherical, ellipsoidal geometry. In this geometry, the degeneracy of the azimuthal modes is lifted, causing the narrow and typically resolution-limited laser peaks to split and broaden into an array of separate peaks. The exact spectral position of the modes is given by the semi-minor and semi-major axes of the droplet, the ratio of the internal and external refractive indices, the angular momentum mode number (*m*), and the azimuthal mode number (*l*). The optical resolution limit of the spectrometer (50 pm) precludes the fitting of the (2*l*+1) separate azimuthal modes which will each have a broadened profile with a minimum width equal to the optical resolution. Furthermore, the azimuthal mode positions do not split evenly, showing a pronounced bunching of lower or higher order modes depending on whether the droplet deforms into an oblate or a prolate ellipsoid, respectively (see Fig. 1b). Consequently, it is not possible to establish the exact azimuthal mode positions. Instead, we look at the splitting of the modes, e.g. the difference between the highest and lowest azimuthal mode which provides sufficient information to reconstruct the geometry of the ellipsoid.

To use this approach the data is prepared by cropping the spectrum into the 4 subsequent split peaks that represent 2 subsequent pairs of TE and TM modes. Next, the background is subtracted from the cropped spectrum using a linear fit to the baseline. The width of each split peak is then found at the position where the intensity of the left and right edge of the peak reaches a level of about 10% of the maximum peak intensity.

### Force analysis

The force analysis uses a maximum error approach which utilises the maximum boundary conditions at each stage of the analysis to estimate the force applied to the droplet within a maximum error margin. We first approximate the size of the ellipsoid from the microlaser spectrum using the following equation,

2 $$a=\frac{{\lambda }_{l}{\lambda }_{l+1}}{2\pi {n}_{\text{eff}}{{FSR}}_{l,l+1}}$$

where *λ*_*l*_ and *λ*_*l*+1_ are the spectral positions of either the lowest or highest azimuthal mode of 2 subsequent split peaks of the same polarization with angular momentum mode number *l* and *l* + 1, *n*_eff_ is the effective refractive index which is the average refractive index that the modes travel in, ${{FSR}}_{l,l+1}$ is the free spectral range between angular momentum modes *l* and *l* + 1, *a* is the semi-axis of the equatorial plane of the ellipsoidal microlaser. Averaging the difference of both the leading and trailing edges (corresponding to the highest and lowest order azimuthal modes) of neighboring angular momentum modes will provide an accurate value of the free spectral range of the resonator. Knowing that the deformation of the ellipsoid is overall very small (the difference in the major axes of the ellipsoid is <1% of the diameter of the undeformed, spherical microlaser), this first analysis gives a very good approximation for the overall size of the microlaser as well as for the angular momentum mode numbers.

The effective refractive index *n*_eff_ is defined using prior knowledge of the maximum external refractive indices coupled with an estimation of the maximum amount of evanescent field that will be traveling within the external refractive index. For this assumption, we can assume that the highest possible effective refractive index will be when the entire mode is contained within the microlaser (*n*_eff_ = *n*_laser_) and the lowest refractive index will occur when there is about 30% of the mode traveling in water (evanescent field component), for which ${n}_{\text{eff}}=0.7{n}_{\text{laser}}+0.3{n}_{\text{water}}$. Water is assumed to be the external refractive index because in all biological experiments presented here the external refractive index will be equal to or greater than water. Now, for a microlaser spectrum consisting of four split peaks, there are 4 possible instances of finding the FSR, using either the leading or trailing edge of each TE and TM mode pair. To find the upper and lower bounds of *a*, we use Eq. (2) with each combination of the FSR and the bounds of the effective refractive index. Using the maximum error approach, the bounds of *a* will be the minimum (*a*_min_) and maximum (*a*_max_) value of *a* found evaluating all combinations of effective refractive index and FSR.

To determine the polar semi-axis, we make a first estimation by calculating the maximum deformation of the polar semi-axis that can occur before the peaks of adjacent TE and TM modes start to overlap. For this, we calculate the highest (*p* = *l*) and lowest (*p* = 0) azimuthal mode from the following equation^30^:

3 $$\begin{array}{l}{\rm{\lambda }}=2\pi {n}_{\text{laser}}a{\left[l-{\alpha }_{q}{\left(\frac{l}{2}\right)}^{\frac{1}{3}}+\frac{2p\left(a-b\right)+a}{2b}-\frac{\chi n}{\sqrt{{n}^{2}-1}}+\frac{3{\alpha }_{q}^{2}}{20}{\left(\frac{l}{2}\right)}^{-\frac{1}{3}}\right.}\\\qquad-\frac{{\alpha }_{q}2{n}^{3}\chi \left(2{\chi }^{2}-3\right)}{12{\left({n}^{2}-1\right)}^{\frac{3}{2}}}{\left(\frac{l}{2}\right)}^{-\frac{2}{3}}-\frac{{\alpha }_{q}2p\left({a}^{3}-{b}^{3}\right)+{a}^{3}}{12{b}^{3}}{\left(\frac{l}{2}\right)}^{-\frac{2}{3}}\\\qquad{\left.+\left(\frac{{\alpha }_{q}^{3}+10}{1400}+\frac{{\left(2p+1\right)}^{2}{a}^{2}\left({b}^{2}\left(1+3{\rm{\mu }}\right)-{a}^{2}\right)}{32{b}^{4}}\right){\left(\frac{l}{2}\right)}^{-1}\right]}^{-1}\end{array}$$

Where $p=l-\left|m\right|$, *b* is the polar semi-axis, *n* is the ratio of internal and external refractive indices, ${\alpha }_{q}$ are the zeroes of the Airy function^30^, *χ* = 1 for TE modes and $\chi =\frac{1}{{n}^{2}}$ for TM modes.

Following this analysis, the maximum deformation that can occur before the peaks start to overlap was found to be 50 nm in the polar semi-axis. Which is valid for droplet diameters ranging from 4 to 100 μm. The initial estimations of the size parameters therefore branch into two separate calculations using *b*_max_ = *a*_max_ ± 50 nm and the other using *b*_min_ = *a*_min_ ± 50 nm as individual starting points. From here the maximum and minimum values of force can be found. To be able to use Eq. (3) to define the size parameters, bounds for the external refractive index need to be defined. The lower bound of the external refractive index is given by the value for water at *n* = 1.333 and the upper bound is *n* = 1.41 within a cell or tissue^49^.

With the bounds for the external refractive index and the upper and lower bounds of the equatorial and polar semi-axes defined, it is possible to use the experimental spectrum to identify a range of possible angular momentum mode numbers. To do this, a spectral look up table is built for angular momentum mode numbers *l*between *l*_min_ = 10 and *l*_max_ = 1000. These bounds are lower and higher, respectively, than the angular mode numbers expected for the ~15 μm diameter microlasers but cover all eventualities as per the maximum error approach. The spectral position of the modes is calculated for each value of external refractive index, equatorial semi-axis and polar semi-axis, also generalizing whether the mode is a TE or TM. Then, each simulated peak is compared to each experimental peak individually and the best fitting angular momentum mode is found for each combination of variables. Using the range of best fitting angular momentum mode numbers, the highest and lowest mode numbers are propagated forward in the analysis as the upper and lower bounds for the angular momentum mode number.

With the upper and lower bounds of *a*, *n*, and *l* found, the last parameter to improve upon is the polar semi-axis or eccentricity of the ellipsoid as we have still used the initial estimation in the analysis until now. To refine the size of the polar semi-axis, a similar process as was used for the angular momentum mode numbers is applied. For each value of the equatorial semi-axis the corresponding polar semi-axis range (±50 nm) is split into a linearly space array of 10,000 elements (10 pm steps). Then, for each combination of equatorial semi-axis, polar semi-axis array, external refractive index and angular momentum mode numbers, the spectral position of the azimuthal modes is calculated using Eq. (3). The value of the polar and equatorial semi-axis that best fits the splitting between the highest and lowest azimuthal mode positions of the experimental peak is used to calculate the eccentricity *e*. The best fitting polar semi-axis is matched to the experimental peak twice as the dataset covers oblate and prolate geometries. The minimum and maximum values of *e* across all the combinations of variables are recorded. This process is repeated for every split peak in each spectrum. All *e* values for every split peak in the spectrum are then compared, and the minimum and maximum values are taken forward for the calculation of the force.

Finally, the force causing the deformation of a spherical droplet into an ellipsoidal droplet with eccentricity *e* is calculated using Eq. (1). By also using the surface tension of the droplet microlaser measured with the pendant droplet tensiometer (Supplementary Fig. S1). Here, the mean value of the *e*^2^-range and the mean value of the *a*-range is used for calculating the force, while the difference from the middle to each bound is used to estimate the error in the force. Consequently, the value of the mechanical force that is calculated here represents a mean force that does not differentiate whether the ellipsoid is in an oblate and prolate geometry and the error is a cumulation of maximum uncertainties.

### Visualization of azimuthal WGMs

Azimuthal WGMs were visualized by plotting either the 2D cross-section of the WGM or the real component of the radial electric field distribution on the surface of the resonator, employing solutions to Maxwell’s equations outside a dielectric sphere as proposed by Oraevsky^50^. To show the evolution of the mode shape, three modes are plotted (Fig. 1a). For *m* equals *l*, the electric field is concentrated almost entirely in the equatorial plane. However, for *m* < *l* the mode exhibits $l-\left|m\right|$ number of nodes along the azimuthal axis. Moreover, as *m* approaches zero, the maximum of the electric field shifts increasingly towards the poles. All calculations were performed in MATLAB using a custom written code.

### Supplementary information

Supplementary information

Supplementary video 1

Supplementary video 2

## Data Availability

The research data supporting this publication can be accessed at 10.17630/a60a63e0-11a1-4ff9-b54d-699a7f0b3dd3.
