# Supplementary material for: Deformable microlaser force sensing
Source: Light Sci Appl. 2024 Jun 5;13:129. doi: 10.1038/s41377-024-01471-9 (PMC11150448; doi:10.1038/s41377-024-01471-9)
Supplement: Supplementary file 1 — Supplementary information [file 41377_2024_1471_MOESM1_ESM.pdf]

Supplementary information for

Deformable microlaser force sensing

Eleni Dalaka[1,4°], Joseph S. Hill[1,3°], Jonathan H. H. Booth[2,3], Anna Popczyk[3], Stefan R. Pulver[2], Malte C. Gather* [1,3], Marcel Schubert* [3]

[1] Centre of Biophotonics, SUPA, School of Physics and Astronomy, University of St Andrews, North Haugh, St Andrews, UK

[2] School of Psychology and Neuroscience, University of St Andrews, St Mary's Quad, South Street, St Andrews, UK

[3] Humboldt Centre for Nano- and Biophotonics, Department of Chemistry, University of Cologne, Köln, Germany

[4] Current address: Institute for Bioengineering of Catalonia, Barcelona, Spain

° These authors contributed equally

E-mail: malte.gather@uni-koeln.de, marcel.schubert@uni-koeln.de

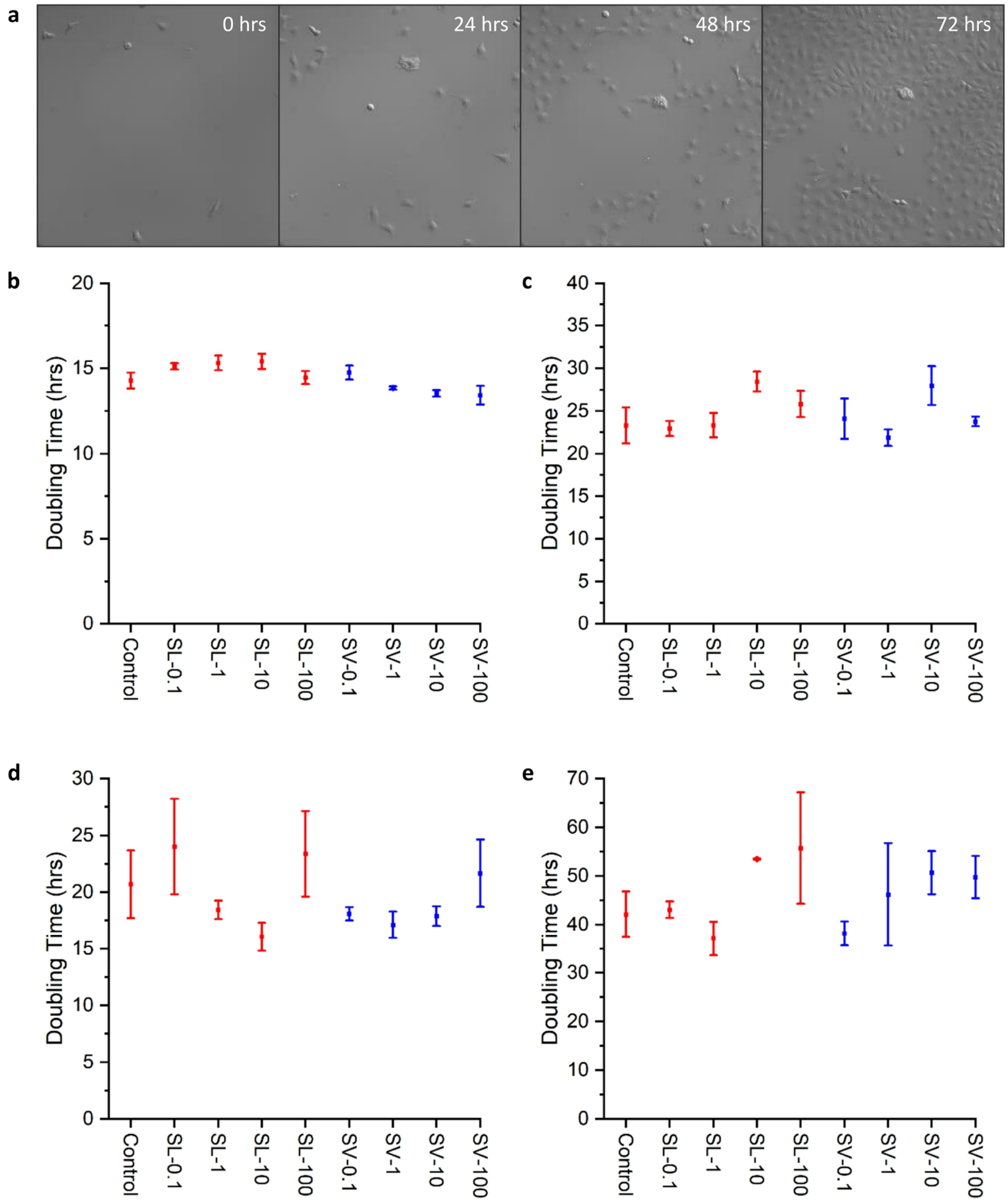

Figure S1 | Assessing droplet microlaser biocompatibility. a. Example differential interference contrast microscopy images from a cell proliferation assay in which the doubling times of primary rat aortic smooth muscle cells (RASMC) was measured over 3 days. Four different cell types were tested, including **b.** RASMC, **c.** 3T3-NIH cells, **d.** primary small intestine mouse endothelial cells (SIMEC), and **e.** primary human umbilical endothelial cells (HUVEC). The tested combinations are labelled as "Oil type-surfactant concentration", and include the two oils Santolight SL5267 (SL) and Santovac 5 (SV) while the concentration of the surfactant Tween 20 is given in multiples of the CMC.

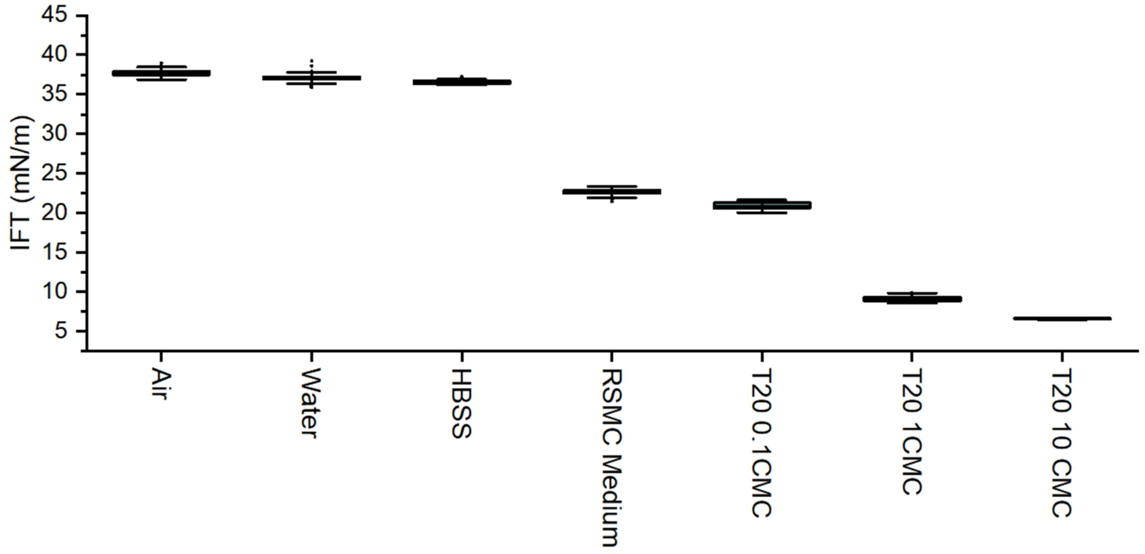

Figure S2 | **Measurement of surface tension with a pendant droplet tensiometer.** Calculated interfacial tension (IFT) of pendant droplets in the following media: air, water, bufferred saline solution (HBSS), rat aortic smooth muscle cell medium, and Tween 20 (T20) solutions at 0.1, 1, and 10 times the critical micelle concentration (CMC). The CMC for T20 is equal to 60 µM.

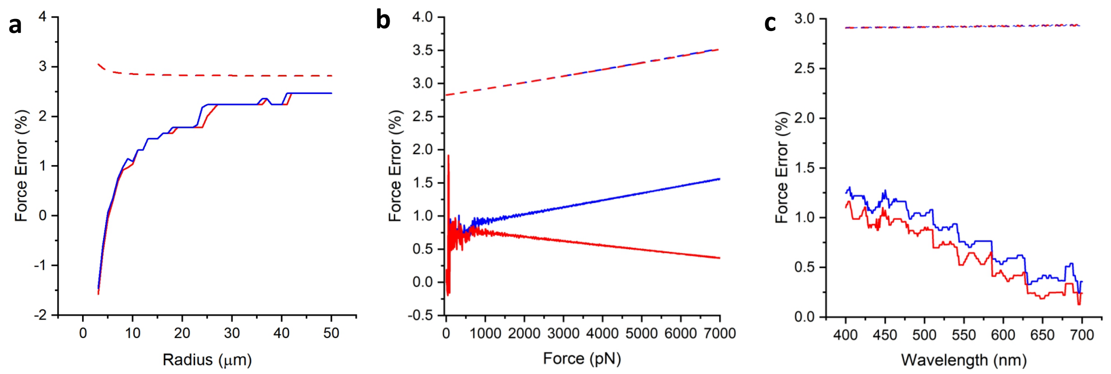

Figure S3 | Testing the accuracy of the maximum error propagation model developed for this study. First, theoretical mode splittings were calculated assuming a certain force and surface tension and by using the analytical model described in methods part of the main text (see Eq. 3) and the following parameters: $n_{\mathrm{Laser}} = 1.63$, $n_{\mathrm{Cell}} = 1.38$, $\gamma = 37\ \mathrm{mN\ m^{-1}}$, $\lambda_{\mathrm{ref}} = 540\ \mathrm{nm}$. The simulated spectra were then fed into the analysis software and the calculated force is compared to the value used in the calculation of the mode splitting. The force errorr for oblate (solid red lines) and prolate (solid blue lines) spheroids were determined for **a.** the radius of the droplets ranging from 3 to 50 µm at a force of 500 pN, **b.** the force ranging from 7 pn to 7 nN for a droplet with a radius of 7.5 µm, and **c.** for the reference wavelength of the spectrum for a droplet with a radius of 7.5 µm at a force of 1 nN. The model also estimates an error for the calculated forces which is displayed as dashed lines. In the large paramter space shown here, this error is typically about 3% and always larger than the actuall error. The force errors provided for the measurments in the main text therefore represent a conservative estimation.

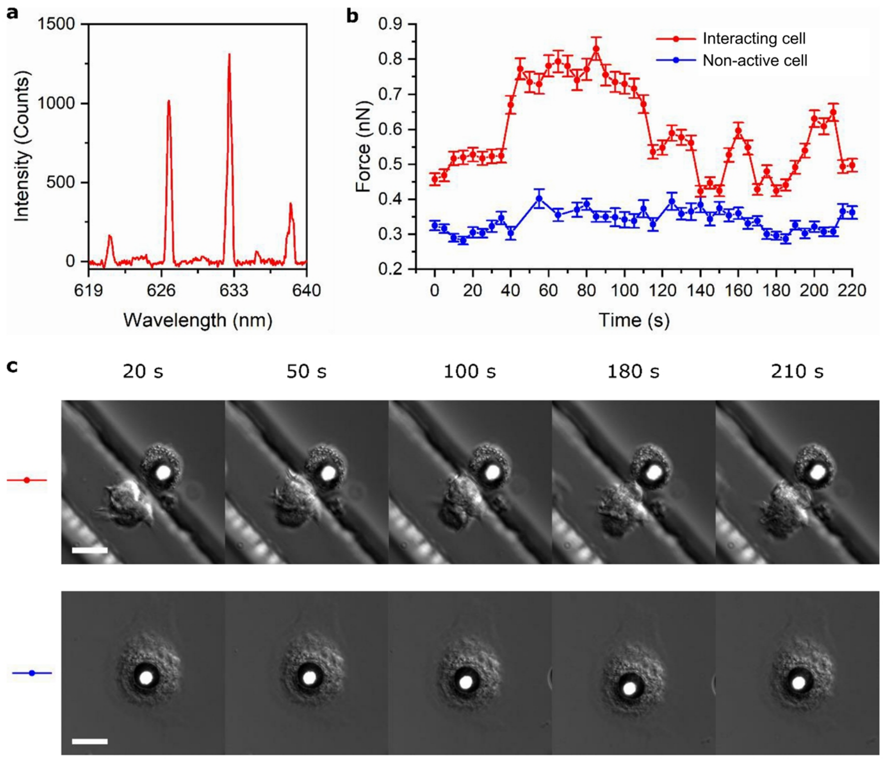

Figure S4 | Measurement of intracellular forces. a. Spectrum of a droplet microlaser internalised by a dendritic cell. **b.** The force exerted on the droplet over time for two cells as calculated from the splitting of the lasing peaks. The interfacial tension of the droplets was assumed to be 4.5 $\mathrm{mN\ m^{-1}}$. Two phenotypes were observed in the culture. Interacting cells (red curve) were migrating and occasionally interacting with other cells, while non-active cells (blue curve) did not migrate and were stationarry during the course of the experiment. **c.** DIC images of an interacting (top) and non-active (bottom) cell at different time points throughout the measurement. Scale bars, 20 µm.

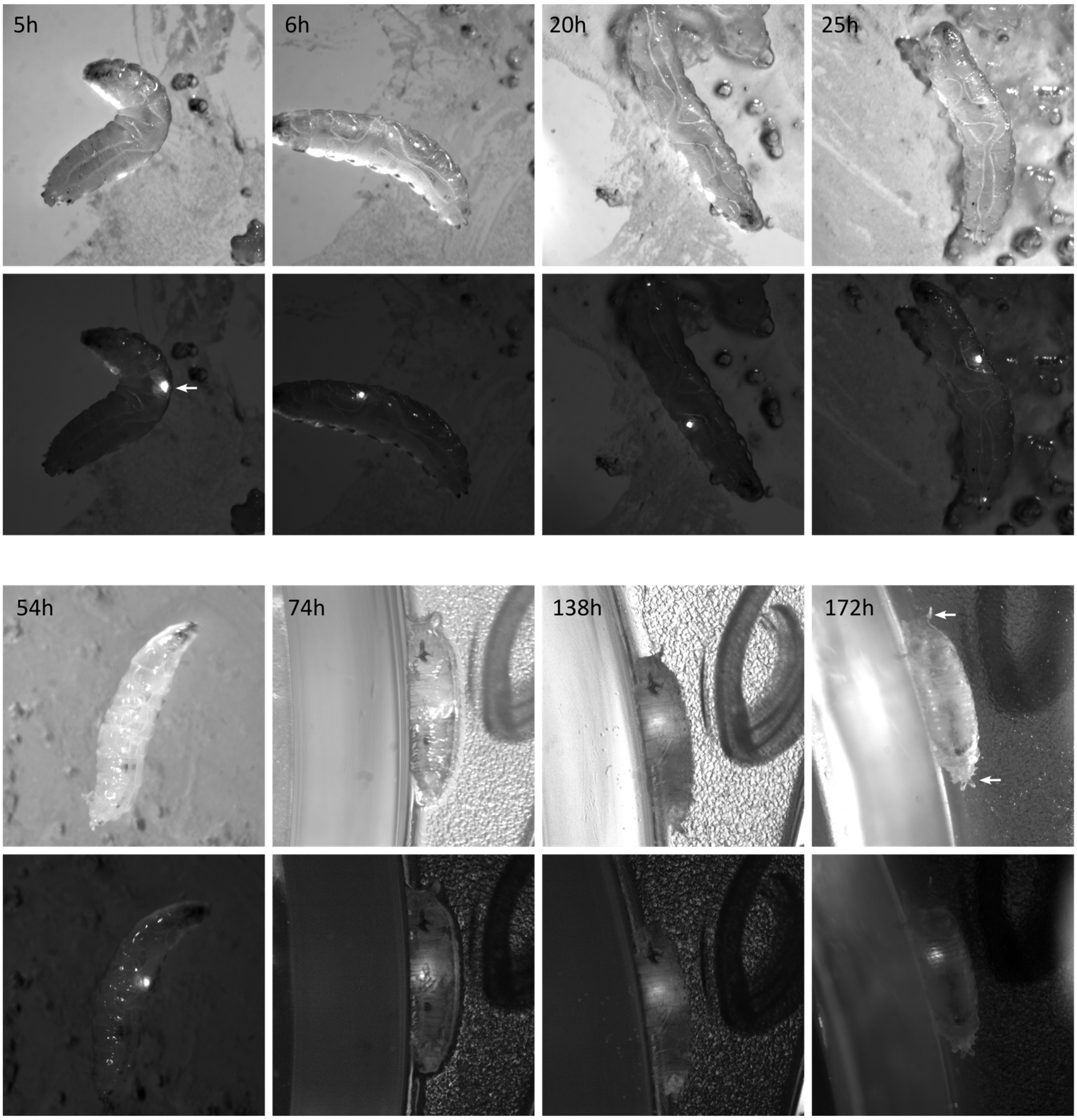

Figure S5 | Tracking of a droplet microlaser inside a *Drosophila* melanogaster larva. Transmission microscopy (top) and fluorescence microscopy (bottom) images of a single *Drosophila* larvae at 8 different time points. The starting point of the experiment was the injection of the droplet and the time is displayed as time after injection. The injected droplet microlaser is indicated by the white arrow in the first frame. The experiment covers the larva and pupa phase. The microlaser can be traced during the entire experiment, although the emission becomes much weaker and more diffuse in the pupa phase. The continued development during the pupa phase can be seen by the appearance of distinct anatomical features highlighted by the white arrows in the last frame. Transmission and fluorescence images were taken with a delay of about 2 to 5 seconds.

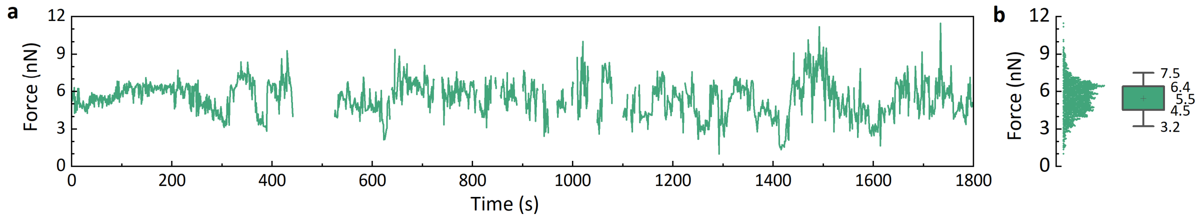

Figure S6 | Continuous force measurements in *Drosophila* melanogaster larvae. a. Force calculated form the laser spectra shown in Fig. 4b (main text). **b.** Descriptive statistics of the force transient shown in (a).

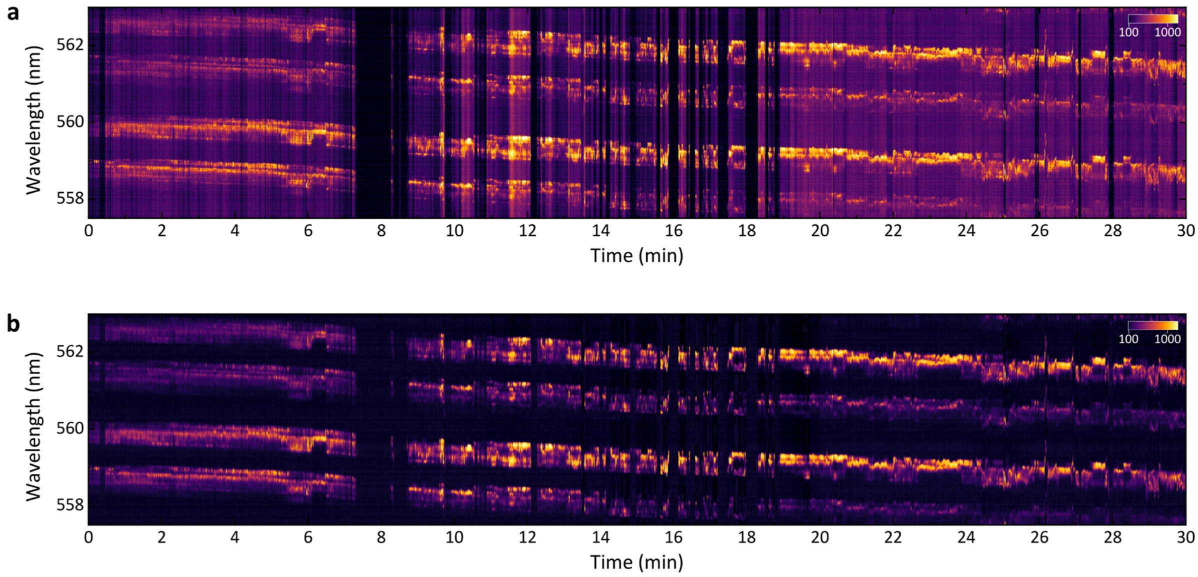

Figure S7 | **Continuous force measurements in *Drosophila* melanogaster larvae. a.** Raw and **b.** processed data (identical with data shown in Fig. 4b, main text). A low level Savitzky-Golay filter was applied to smooth the laser spectrum at each time point. In addition, due to large intensity variations during the 30 min measurement all spectra were normalized to their average background intensity (measured in between two adjacent WGMs where no lasing emission is present). Dark vertical bands represent time points where the droplet laser moved out of the pump spot caused by the internal tissue movements of the animal.
